# Frequent alterations in p16/*CDKN2A* identified by immunohistochemistry and FISH in chordoma

**DOI:** 10.1002/cjp2.156

**Published:** 2020-01-08

**Authors:** Lucia Cottone, Nadia Eden, Inga Usher, Patrick Lombard, Hongtao Ye, Lorena Ligammari, Daniel Lindsay, Sebastian Brandner, Jože Pižem, Nischalan Pillay, Roberto Tirabosco, Fernanda Amary, Adrienne M Flanagan

**Affiliations:** ^1^ UCL Cancer Institute University College London London UK; ^2^ Department of Histopathology Royal National Orthopaedic Hospital Stanmore UK; ^3^ UCL Queen Square Institute of Neurology University College London London UK; ^4^ Division of Neuropathology, The National Hospital for Neurology and Neurosurgery University College Hospitals NHS Foundation Trust London UK; ^5^ Institute of Pathology University of Ljubljana, Faculty of Medicine Ljubljana Slovenia

**Keywords:** chordoma, copy number, p16, *CDKN2*A, IHC, FISH, biomarker

## Abstract

The expression of p16*/CDKN2A*, the second most commonly inactivated tumour suppressor gene in cancer, is lost in the majority of chordomas. However, the mechanism(s) leading to its inactivation and contribution to disease progression have only been partially addressed using small patient cohorts. We studied 384 chordoma samples from 320 patients by immunohistochemistry and found that p16 protein was lost in 53% of chordomas and was heterogeneously expressed in these tumours. To determine if *CDKN2A* copy number loss could explain the absence of p16 protein expression we performed fluorescence *in situ* hybridisation (FISH) for *CDKN2A* on consecutive tissue sections. *CDKN2A* copy number status was altered in 168 of 274 (61%) of samples and copy number loss was the most frequent alteration acquired during clinical disease progression. *CDKN2A* homozygous deletion was always associated with p16 protein loss but only accounted for 33% of the p16‐negative cases. The remaining immunonegative cases were associated with disomy (27%), monosomy (12%), heterozygous loss (20%) and copy number gain (7%) of *CDKN2A*, supporting the hypothesis that loss of protein expression might be achieved via epigenetic or post‐transcriptional regulatory mechanisms. We identified that mRNA levels were comparable in tumours with and without p16 protein expression, but other events including DNA promoter hypermethylation, copy number neutral loss of heterozygosity and expression of candidate microRNAs previously implicated in the regulation of *CDKN2A* expression were not identified to explain the protein loss. The data argue that p16 loss in chordoma is commonly caused by a post‐transcriptional regulatory mechanism that is yet to be defined.

## Introduction

Chordoma is a rare primary malignant bone tumour showing notochordal differentiation and affected individuals have a median survival of 7 years from presentation 1, 2. Chordoma is characterised by expression of the embryonic transcription factor *TBXT*, also known as *brachyury* and *T*, which plays a critical role in the development of the disease 3, 4. Genomic studies have failed to identify recurrent genetic driver alterations other than copy number gain of *TBXT* in 27% of cases 5 in addition to occasional sporadic chromosomal rearrangements and alterations involving *RB1*, *TP53* and cyclin dependent kinase inhibitor 2A (*CDKN2A*) 6.

The *CDKN2A* gene (chromosome 9p21) encodes the proteins p14^ARF^ and p16^INK4a^, also referred to as p16, generated through alternative exon usage 7. p16 is transcribed using exons 1α, 2 and 3, whereas p14^ARF^ is transcribed using exon 1β and exon 2. Both proteins are involved in cell cycle control via the Rb and p53 pathways which are critical for self‐renewal and ageing 8. p14^ARF^ stabilises and activates the p53 pathway, whereas p16 blocks G1/S cell cycle progression by preventing phosphorylation of Rb: disruption of control of these pathways plays a pivotal role in the progression of a variety of cancers 9. *CDKN2A* is part of a locus that also contains *CDKN2B*, which encodes p15^INK4b^, a tumour suppressor that, like p16^INK4a^, inhibits CDK4/CDK6 10.

*CDKN2A* is the second most frequently inactivated tumour suppressor gene in cancer 9, 11 and its inactivation is achieved in the majority of cases via homozygous deletion or promoter hypermethylation 11. Germline mutations in *CDKN2A* confer susceptibility to melanoma and other tumours 12, 13, and haploinsufficiency of p14^ARF^ has been implicated in genetic models of various cancers 12, 14.

The *CDKN2A* gene locus is deleted and p16 protein expression is lost in a number of chordoma cell lines 15, 16. Loss of p16 protein expression has also been reported in up to 80% of chordomas 6, 17, 18. The mechanism leading to its inactivation and the contribution of *CDKN2A* loss to disease progression have only been partially elucidated. Using small numbers of chordoma samples, it has previously been reported that 3–33% of chordoma cases harbour homozygous deletions of *CDKN2A* 6, 17, single nucleotide variations are rare 5, 18, and DNA promoter hypermethylation is an uncommon event 6. The absence of therapeutic options for patients with chordoma makes this observation clinically significant as p16 loss has been shown to sensitise to CDK4/6 inhibitors 15, 19, 20, making expression of p16 a potential biomarker for patient stratification and prognosis. This prompted us to interrogate a large number of chordoma samples with the aim of increasing our understanding of the role of *CDKN2A* inactivation in the pathogenesis of chordoma.

## Materials and methods

### Chordoma samples

Tumour diagnoses were made using the WHO classification 2. Frozen tumour material was available for 35 chordomas: 10 were analysed by whole genome sequencing and RNA sequencing and 26 by whole exome sequencing, the results of which have been reported previously 5. Formalin‐fixed paraffin‐embedded samples were obtained from the archive of the Royal National Orthopaedic Hospital and several other sites. The samples were used to construct tissue microarrays (TMAs), which were built as previously described 21.

Ethical approval for in‐house chordoma samples was obtained from the Cambridgeshire 2 Research Ethics Service (reference 09/H0308/165) (HTA Licence 12198). Samples were also obtained through the Brain UK Biobank (reference 14/006 – Large scale genetic and epigenetic screen of chordoma).

### Chordoma cell lines

UCH‐1, UCH‐2, MUG‐Chor, UM‐Chor, UCH‐11, JHC7 (http://www.chordomafoundation.org/) and UCH‐7 16 are well characterised human chordoma cell lines; all derived from sacral tumours except UM‐Chor which was generated from a clival chordoma. U2OS (ATCC® HTB96™, ATCC, Manassas, VA, USA), an osteosarcoma cell line that lacks expression of *TBXT*, used as a control, was cultured according to ATCC guidelines. Cell lines were quality controlled by short‐tandem‐repeat analysis (DNA Diagnostic Centre, London, UK) and were regularly tested to ensure that they were mycoplasma‐free.

### Fluorescence *in situ* hybridisation and immunohistochemistry

Fluorescence *in situ* hybridisation (FISH) was performed as described previously 22 using the *p16/CDKN2A* (9p21) (Vysis, Abbott Molecular, Abbott Park, IL, USA) and the *TBXT* (*Custom probe to chr6:166526346–166 623 395*; Agilent Technologies, Santa Clara, CA, USA) probes. Assessment of *CDKN2A* and *TBXT* FISH was undertaken as previously reported 22: for a probe signal to be counted as abnormal at least 15% of the nuclei analysed were required to reveal an aberrant signal on counting a minimum of 50 consecutive non‐overlapping nuclei. The following categories were determined as follows (1) monosomy (one *p16/CDKN2A* and one centromeric signal); (2) heterozygous deletion (loss of one copy of *p16/CDKN2A* in the presence of two centromeric signals); (3) homozygous deletion (loss of two copies of *p16/CDKN2A* in the presence of one or two centromeric signals) and (4) amplification (*p16/CDKN2A* centromeric ratio greater than 2).

Immunohistochemistry (IHC) was performed on a Leica Bond 3 as previously described 21. The p16 (JC8) antibody (Santa Cruz, USA, catalogue number SC‐56330) was used at a dilution of 1 of 200. This antibody was previously validated by knock‐down *in vitro* experiments 23. As TMAs are not fully representative of heterogenous tumours, IHC was repeated and validated on full sections in samples where there was loss of immunoreactivity: this provided a high concordance (88%, 5 false negatives/43). For those cases for which the results obtained using TMAs was inconclusive, the IHC and FISH were repeated on full tissue sections.

### Real‐time quantitative PCR

Quantitative real‐time PCR (qPCR) for mRNA and miRNA expression was performed as previously reported 16 (see supplementary material, Supplementary Materials and Methods).

### Genomic and transcriptomic analysis

The variant calling pipeline of the Cancer Genome Project at the Wellcome Trust Sanger Institute was used to call somatic mutations. The following algorithms, with standard settings, and no additional post‐processing were used on aligned DNA BAM files: CaVEMan (1.11.0) for substitutions 24; Pindel (2.1.0) for indels 25; BRASS (5.3.3; https://github.com/cancerit/BRASS) for rearrangements, and ASCAT NGS (4.0.0) for copy number aberrations 26. Sequenced RNA libraries were aligned with hisat2 (v2.1.0) to hg19 reference genome. EdgeR (v3.24.0) was used to count gene features.

### DNA methylation analysis

DNA methylation array data (450K or EPIC array, Illumina, CA, USA) of 35 chordoma samples have been published previously 27 and EPIC array was performed on chordoma (UCH1, UM‐Chor, UCH7, MUG‐Chor) and U2OS cell lines. Nucleic acid was prepared as previously reported 5.

### Statistical analysis

Continuous variables were compared via unpaired *t*‐test, whereas categorical variables were compared via Fisher's exac*t* test and Chi Squared test using Wizard 1.9.21. Data were considered statistically significant when *p* < 0.05. *Q* values were calculated using the R package *q* value. In gene expression studies, data were judged to be statistically significant when *P* value was calculated as less than 0.05 by two‐tailed Student's *t*‐test. Statistical analysis was performed in GraphPad PRISM 5.0 (GraphPad Software, La Jolla, CA, USA).

## Results

A total of 384 sporadic chordoma samples were collected from 320 patients from across Europe, including the UK, 286 of which were primary tumours, 86 local recurrences and 12 from metastatic disease. Samples (*n* = 2–6) from more than one time point were studied for 39 patients. The tumours were located in the skull base (*n* = 90), mobile spine (*n* = 48), sacrum/coccyx (*n* = 178) and extra‐axial sites (*n* = 4). The age at presentation of the patients ranged from 6 to 91 (median 60) years: those with tumours located at the skull base, mobile spine, sacro‐coccygeum and at extra‐axial sites presented between the ages of 6–68, 14–79, 14–76 and 24–28 years of age respectively. The female to male ratio was 1:1.53 (83 females, 127 males).

### Genome sequencing data

Whole genome sequencing previously reported 5 revealed copy number alterations at the *CDKN2A* locus in 7 of 10 chordomas studied (see supplementary material, Table S1 and Figure S1). The patterns of *CDKN2A* deletion were variable: three cases showed complete loss of chromosome 9, two cases showed loss of several megabases and one case harboured a smaller deletion in the range of kilobases. One case revealed *CDKN2A* copy number gain; none of the samples showed single nucleotide variants (SNVs) or indels of *CDKN2A*. FISH revealed *TBXT* amplification in three cases: this did not correlate with a specific *CDKN2A* copy number status. Whole genomes and whole exomes (*n* = 35) were also analysed for the presence of copy number neutral loss of heterozygosity (LOH), which was identified in only one case where it was associated with retention of p16 expression in the absence of mutations.

### p16/*CDKN2A* IHC and FISH

**Figure 1:**
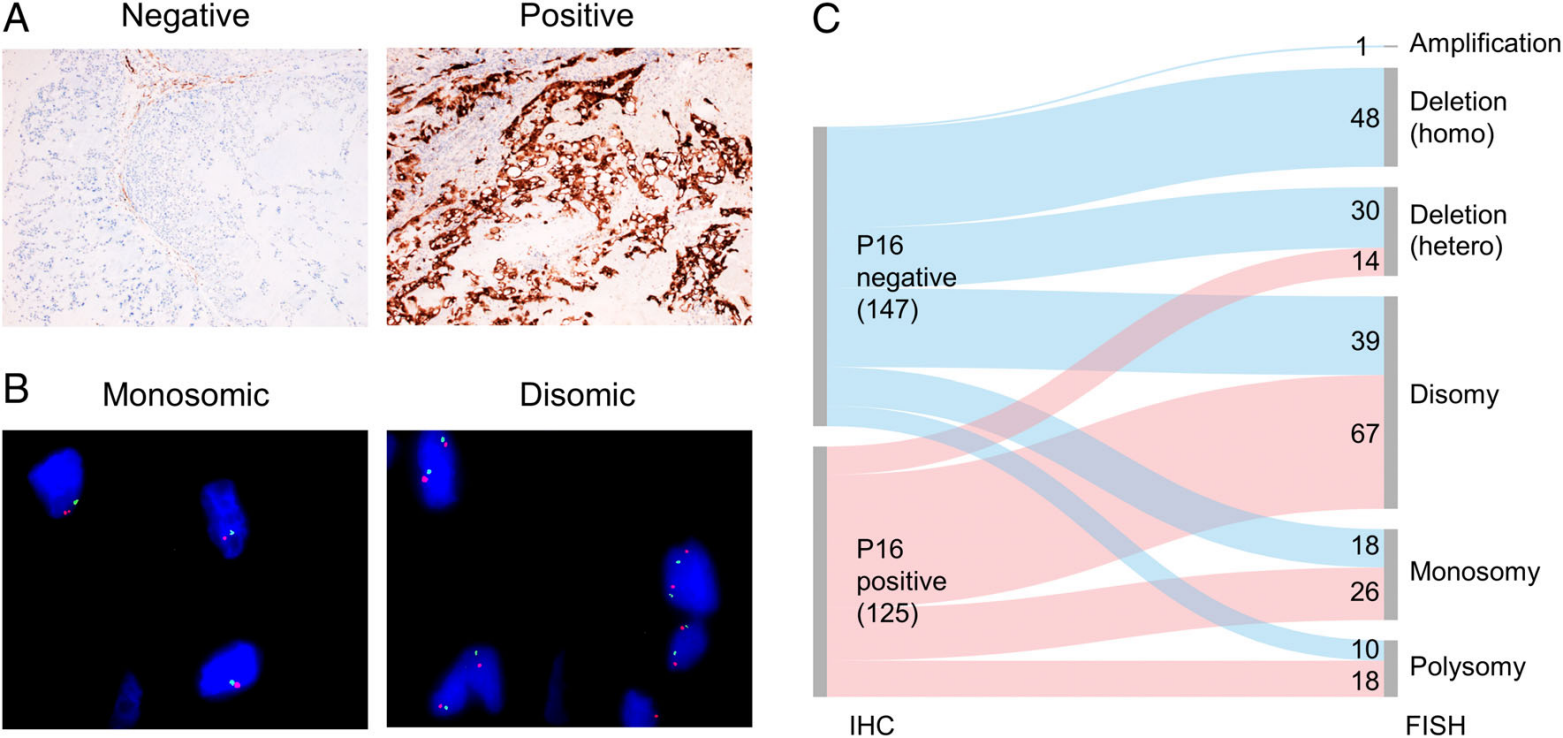
p16 IHC and *CDKN2A* FISH in chordoma samples. (A) Representative IHC images of chordoma cases negative (left) or positive (right) for p16. Objective magnification ×4. (B) Representative images of chordoma cases showing monosomic (left) or disomic (right) copy number status as assessed by FISH. Objective magnification ×100. (C) Sankey diagram of the IHC and FISH results. Results were available for both p16/*CDKN2A* IHC and FISH for at least one sample from 243 of 320 patients.

The result of p16 IHC performed on 303 informative samples demonstrated loss of expression in 53% (162/303) of cases (Figure 1A and Table 1), a finding not dissimilar to previous reports 6, 15. To determine if *CDKN2A* copy number loss could explain the absence of p16 protein expression we performed FISH on consecutive tissue sections to those used for IHC (Figure 1B and Table 1). Results from 274 of these samples were informative: the majority (167/274, 61%) of chordomas harboured *CDKN2A* copy number alterations, the most frequent event being copy number loss which was detected in 138/167 samples (83%) (Table 1).

**Table 1:** p16 IHC and *CDKN2A* FISH data for all informative samples from 243 patients

p16 status (IHC)		*CDKN2A* copy number (FISH)			
	*n* (%)		*n* (%)		*n* (%)
Positive	141 (47)	Normal		Disomy	107 (39)
Negative	162 (53)	Copy number loss	138 (50)	Monosomy	46 (17)
Total	303			Deletion (hetero)	44 (16)
				Deletion (homo)	48 (17)
		Copy number gain	29 (11)	Amplification	1 (1)
				Polysomy	28 (10)
				Total	274

Results were available for both p16/*CDKN2A* IHC and FISH for at least one sample from 243 of 320 patients. Copy number loss is represented by monosomy, heterozygous deletion, or homozygous deletion. Copy number gain is represented by amplification or polysomy.

On aligning the FISH with IHC data (Figure 1C), we found 100% correlation between homozygous loss of *CDKN2A* and p16 loss of expression. However, only 33% (48/147) of the p16 immunonegative samples revealed a *CDKN2A* homozygous deletion, leaving an explanation to be found for the loss of protein expression for the remaining 67% of samples. Notably, among p16 immunonegative cases, 27, 12 and 20% showed disomy, monosomy and heterozygous loss respectively (Figure 1C). This apparent discordance between the FISH and IHC results raises the possibility that protein loss might be achieved via either epigenetic or post‐transcriptional regulatory mechanisms.

### DNA methylation

To determine if an epigenetic mechanism could explain the p16 protein loss in some chordomas, DNA promoter methylation status of *CDKN2A* was assessed in four chordoma cell lines (all p16‐negative) and 35 chordoma samples, 15 of which were negative for p16 immunoreactivity. We found low levels of methylation in all samples, with the exception of the single clival INI‐1‐negative poorly differentiated chordoma analysed and the clival‐derived INI‐1‐negative UM‐Chor cell line: both of these showed higher levels of promoter methylation compared to all other samples, including a second INI‐1 immunoreactive clival tumour and three INI‐1‐positive cell lines (UCH1, MUG‐Chor and UCH7) (see supplementary material, Figure S2A). This demonstrates, particularly when previous reports are considered 6, that DNA methylation rarely accounts for p16 protein loss in chordoma.

### Polymorphism in *CDKN2A*

*CDKN2A* gene expression has been reported to be influenced by the SNP rs11515, which is the most frequent *CDKN2A* polymorphism located in the 3′UTR of the gene and has been associated with various cancers 28. Analysis of whole genomes and exomes revealed that, of 35 cases, 24 (68%) were homozygous (CC genotype) and 11 (32%) were heterozygous (CG genotype) for the major allele. This frequency is similar to that found in the general population 28 thereby excluding an association of the SNP rs11515 with chordoma. Moreover, this genotype did not correlate with p16 loss at the protein level (33% [3/9] CG cases, 60% [12/19] CC cases, *p* = 0.22), nor with *CDKN2A* genetic deletion (22% [4/14] CC cases, 30% [3/10] CG cases, *p* = 0.99). Analysis of RNA sequencing showed that transcriptional levels of p14^ARF^, p16^INK4a^ and ANRIL (a long noncoding antisense transcript part of the *CDKN2A* locus which promotes *CDKN2A* transcriptional repression 29), were also not influenced by the SNP (see supplementary material, Figure S2B–D). Taken together these results argue against the SNP rs11515 influencing p16 expression in chordoma.

### *CDKN2A* gene expression and IHC

**Figure 2:**
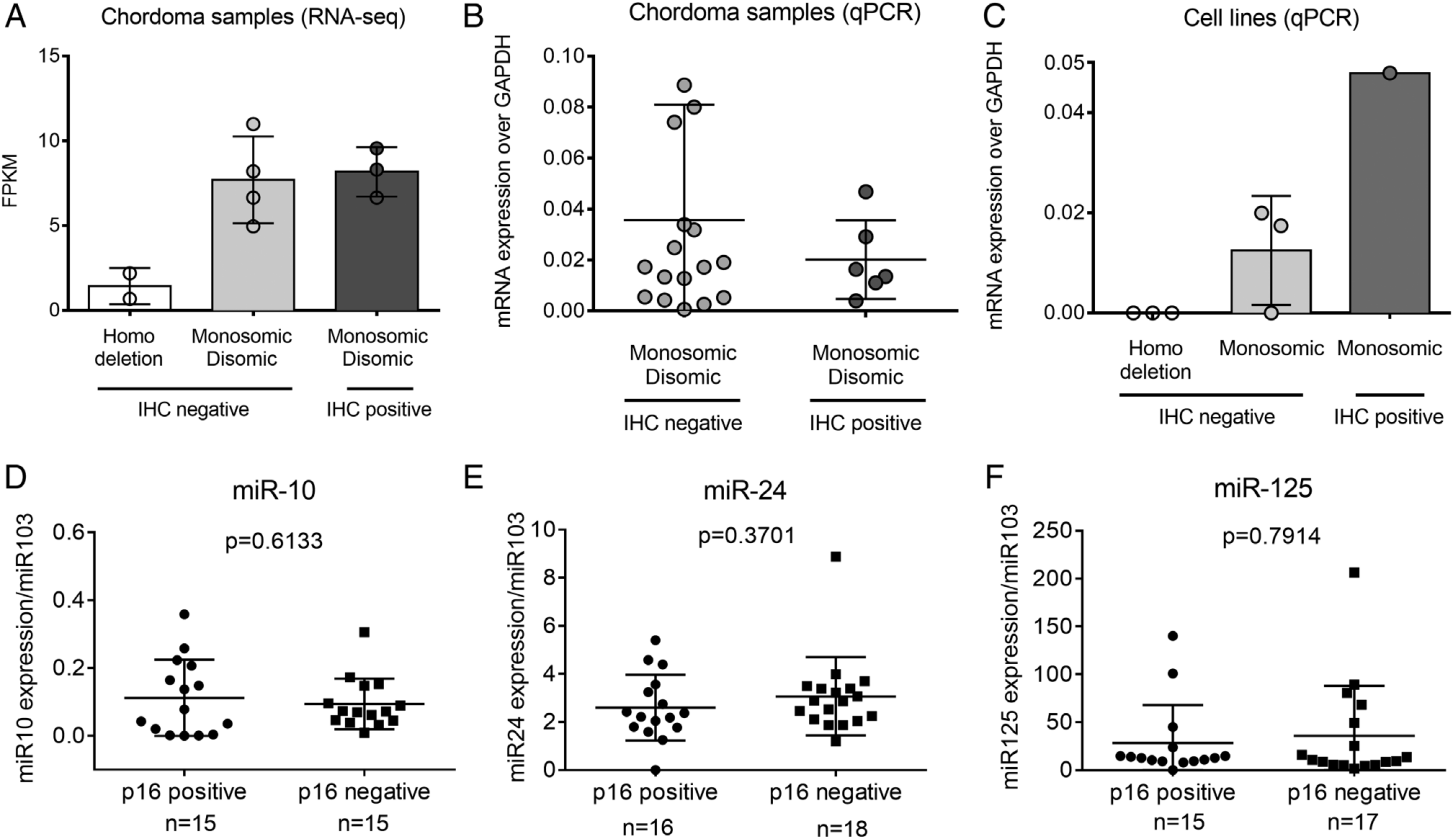
*CDKN2A* transcript levels detected despite p16 immunonegativity in chordoma samples and cell lines and lack of correlation to miRNA expression. (A) Expression of *CDKN2A* transcript assessed in 10 chordoma samples by RNA‐sequencing. FPKM, fragments per kilobase of transcript per million mapped reads. (B) Expression of *CDKN2A* transcript assessed in 22 chordoma samples by qPCR. (C) Expression of *CDKN2A* transcript in chordoma cell lines by qPCR: p16‐negative with homozygous deletion (UCH1, UCH2, MUG‐Chor), p16 negative and monosomic (UCH7, UM‐Chor, UCH11), p16‐positive and monosomic (JHC7). (D–F) Expression of miRNA‐10, –24 and –125 in chordoma cases, as assessed by FISH, and showing positivity or negativity for p16 protein expression by IHC. Cases showing monosomic or disomic *CDKN2A* copy number were combined in this analysis.

To study further the loss of p16 protein expression in the absence of homozygous deletion we analysed the gene expression in 10 cases previously subjected to RNA sequencing 5 (see supplementary material, Table S1). Three of these cases, which showed loss of p16 protein expression, revealed monosomy in two of the cases and disomy in the third by FISH; however, the *CDKN2A* transcript levels were comparable to those that retained p16 immunoreactivity (Figure 2A). These data were supported by detectable levels of *CDKN2A* transcript associated with the loss of expression of p16 at the protein level in an additional 15 chordoma cases assessed by qPCR and in seven chordoma cell lines (three with homozygous deletions and four with monosomy) (Figure 2B‐C). This further supports the concept that loss of p16 protein expression is achieved at the post‐transcriptional level.

### Post‐transcriptional regulation of *CDKN2A*

miRNAs including miR‐24‐2 30, 31, miR‐10b‐5p 32 and miR‐125b 33 have been shown to regulate p16 expression at the post‐transcriptional level in various cancers. We therefore tested the expression of these candidate miRNAs in monoallelic (*n* = 16) and disomic (*n* = 18) chordoma cases that had retained or lost p16 immunoreactivity (Figure 2D–F), but we failed to identify a correlation with p16 expression by IHC, suggesting that other mechanisms yet to be identified control the expression of p16 in chordoma.

### Patients with multiple samples

Next, we assessed tumour heterogeneity and evolution with respect to p16 IHC and *CDKN2A* copy number alteration from patients for whom there was more than one sample available including the primary tumour (Table 2). Informative samples from 36 patients revealed that 26 (72%) showed no change in the immunoreactivity status over time. Tumours from five (14%) patients showed loss of p16 over time and, when correlating these results with *FISH* on consecutive tissue sections, we observed a change in copy number in three cases (Table 2). This included a case of a p16‐positive primary tumour with *CDKN2A* disomy which revealed heterozygous loss in two local recurrences while retaining p16 positivity, and a homozygous deletion with p16 loss in a third local recurrence: this demonstrates a step‐wise acquisition of *CDKN2A* inactivation in chordoma evolution (case 212). However, in another four cases (5, 54, 213 and 214), despite the loss of p16 immunoreactivity over time, there was no change in the copy number, which was represented in all cases by either heterozygous loss or monosomy (Table 2).

**Table 2:** p16 IHC and *CDKN2A* FISH of multiple samples from 39 patients

Study unique ID	Anatomical site	Age at dx	Primary	LR1	LR2	LR3	LR4	LR5	Met	Events per patient	IHC over time	FISH over time
5	Mobile spine	53								2	Pos > Neg	Mono > Mono
20	Mobile spine	68								2	Pos > Pos	Mono > Diso
22	Sacrum/coccyx	63								2	Neg > Neg	NI
23	Sacrum/coccyx	72								2	Pos > Pos	NI
39	Sacrum/coccyx	‐								2	Neg > Pos	Disomy > Disomy
42	Sacrum/coccyx	58								2	Neg > Neg	Del Homo > Del Homo
54	Sacrum/coccyx	43								2	Pos > Neg	Del Het > Del Het
56	Sacrum/coccyx	61								2	Neg > Neg	Del Homo > Del Homo
61	Sacrum/coccyx	58								4	Neg > Neg	NI
67	Sacrum/coccyx	54								2	Pos > Pos	Del Het > Del Het
88	Sacrum/coccyx	67								2	Neg > Pos	Mono > Mono
90	Sacrum/coccyx	67								2	Neg > Neg	Del Het > Del Het
95	Sacrum/coccyx	63								3	Neg > Neg	Mono > Mono
131	Sacrum/coccyx	69								2	Pos > Pos	NI
134	Sacrum/coccyx	65								2	NI	NI
135	Sacrum/coccyx	65								2	Pos > Pos	NI
136	Sacrum/coccyx	73								2	Neg > Neg	NI
137	Sacrum/coccyx	69								2	Neg > Neg	NI
138	Sacrum/coccyx	61								3	Neg > Neg	Del Het > Del Het
143	Sacrum/coccyx	‐								6	Pos > Pos	Poly > Poly
153	Sacrum/coccyx	59								2	Pos > Pos	Mono > Mono
157	Sacrum/coccyx	53								2	Pos > Pos	Mono > Poly
162	Skull base	43								3	Pos > Pos	Mono > Poly
165	Mobile spine	49								2	Pos > Pos	NI
166	Sacrum/coccyx	63								2	Neg > Neg	Mono > Mono
168	Skull base	47								3	NI	Diso > Mono
173	Skull base	19								2	NI	NI
174	Skull base	35								2	Pos > Pos	NI
175	Sacrum/coccyx	35								3	Neg > Neg	Del Het > Del Het
177	Skull base	37								3	Pos > Pos	Poly > Poly
182	Skull base	65								2	Pos > Pos	Diso > Diso
209	Skull base	40								4	Pos > Pos	Diso > Poly
211	Skull base	63								3	Neg > Pos	Del Het > Del Het
212	Skull base	46								4	Pos > Neg	Diso > Del het > Del homo
213	Skull base	60								3	Pos > Neg	Diso > Mono
214	Mobile spine	57								2	Pos > Neg	Diso > Del homo
217	Skull base	31								3	Neg > Pos	Mono > Mono
218	Vertebra	53								5	Neg > Neg	Del Homo > Del Homo
219	Mobile spine	48								3	Neg > Pos	Del Homo > Del Het

Blue, samples analysed. p16 IHC was NI for three patients, leaving 36 patients available for analysis. Del Het, Heterozygous deletion; Del Mono, Homozygous deletion; Diso, disomy; Dx, diagnosis; LR1–LR5, first to fifth local recurrence; Met, metastasis; Mono, monosomy; Neg, p16‐negative; NI, non‐informative; Poly, polysomy; Pos, p16 positive.

**Figure 3:**
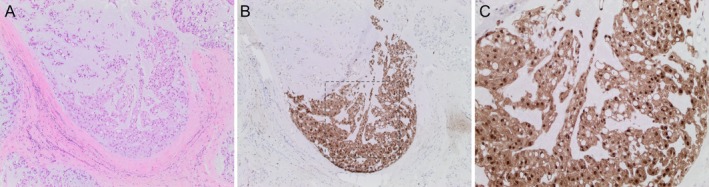
Heterogeneous expression of p16 in chordoma samples. (A–C) Representative IHC images of one chordoma case showing heterogeneous expression of p16: H&E (A, ×4 objective magnification), p16 (B and C, ×4 and ×10 objective magnification respectively). C shows approximately the region enclosed by the dotted lines in B.

Samples from another five patients (39, 88, 211, 217 and 219) revealed loss of p16 expression in the primary tumour but retention in one of the local recurrences (Table 2). We postulated that this could be explained by incomplete excision of an area of the primary tumour which was p16 immunoreactive. This was confirmed when we showed that p16 immunoreactivity was present focally in sections from one of two tissue blocks of the same specimen (case 39) (additional tissue blocks from the other four cases were not available for testing) (Figure 3 and see supplementary material, Table S2). We then tested for p16 expression in multiple blocks from another five cases (22, 56, 61, 90 and 95): three cases were consistently negative whereas two cases showed heterogeneous expression of p16 (see supplementary material, Figure S3 and Table S2). Tumour heterogeneity could therefore explain the finding of the absence of p16 in the primary tumours and the retention in local recurrences (cases 39, 88, 211, 217 and 219). This could also explain the finding of *CDKN2A* monosomy in a primary tumour and disomy (cases 20) or polyploidy (cases 157 and 162) in local recurrences. However, these FISH results could also be accounted for by complex structural alterations developing over time including whole chromosomal doubling, but a detailed analysis using whole genome sequencing would be required to confirm such a mechanism.

### p16/*CDKN2A* status and clinical data

Finally, we correlated *CDKN2A* FISH and IHC results with patients' clinical data (Tables 3 and 4). The anatomical location of the tumour did not influence *CDKN2A* copy number status or p16 protein expression. There was a trend towards *CDKN2A* copy number loss being more common in older patients (>40 years old, *p* = 0.020, *q* = 0.102) and in samples from local recurrences compared to primary tumours (*p* = 0.048, *q* = 0.154). Analysis of the 12 metastatic tumours available revealed that p16 protein expression was lost in all samples (*p* = 0.0005, *q* = 0.005, compared to primary tumours). *CDKN2A* copy number loss in metastatic disease was not significantly different form primary tumours (*p* = 0.225, *q* = 0.342).

**Table 3:** p16 immunoreactivity and clinical information on samples from 243 patients with chordoma

p16 (IHC)		Positive (%)	Negative (%)	*P* value	*q* value
Age	<40	16/22 (72)	6/22 (28)		
	>40	58/119 (49)	61/119 (48)	0.0617	0.1542
Stage of tumour	Primary	103/211 (49)	108/211 (51)		
	LR1–LR5	39/81 (48)	42/81 (52)	0.8960	0.8960
	Metastasis	0/12 (0)	12/12 (100)	0.0005	0.0050
Anatomical Site	Sacrum‐Coccygeal	54/108 (50)	54/108 (50)		
	Mobile Spine	16/26 (62)	10/26 (38)	0.4829	0.5365
	Skull Base	20/57 (35)	37/57 (65)	0.1869	0.3417

Results were available for both p16/*CDKN2A* IHC and FISH for at least one sample from 243 of 320 patients. Age relates to age at the time of diagnosis of the primary tumour. Anatomical site relates to the site at presentation.

**Table 4:** *CDKN2A* copy number by FISH and clinical information for patients with chordoma

*CDKN2A* (FISH)		Disomic (%)	Copy number loss (%)	Copy number gain (%)	*P* value	*q* value
Age	<40	15/22 (68)	6/22 (27)	1/22 (5)		
	>40	44/113 (39)	58/113 (51)	11/113 (10)	0.0204	0.1020
Stage of tumour	Primary	86/193 (44)	88/193 (46)	19/193 (10)		
	LR1–LR5	19/70 (27)	43/70 (61)	8/70 (12)	0.0480	0.1542
Anatomical site	Metastasis	2/11 (18)	7/11 (64)	2/11 (18)	0.2253	0.3417
	Sacrum‐coccyx	36/93 (37)	47/93(52)	10/93 (11)		
	Mobile spine	14/25 (56)	10/25 (40)	1/25 (4)	0.2392	0.3417
	Skull base	28/56 (50)	21/56 (37)	7/56 (13)	0.3342	0.4177

Results were available for both p16/*CDKN2A* IHC and FISH for at least one sample from 243 of 320 patients.

## Discussion

In this p16 IHC study of chordomas, the largest to date, we report that diffuse p16 loss is a frequent finding in this disease, occurring in at least 53% of cases, confirming the previously described frequent loss (66%) in 43 chordoma cases 15. Much of the reported evidence available to date implies that this loss is associated with either a heterozygous or homozygous deletion of the region covering the *CDKN2A* locus but the number of chordoma samples studied using both markers is limited. Specifically, the array CGH findings by Hallor and colleagues 17 showed that 70% of cases displayed genetic alterations but the protein expression was not studied. Le et al 6 performed immunohistochemical analysis on 18 cases and showed loss of expression in 83% of cases; they also observed loss of 9p, either through entire chromosome 9 loss or partial 9p loss alone, in 15/20 cases (75%) by array CGH. Our FISH results also corroborate the published array CGH findings, with 50% of chordomas revealing copy number loss represented by homozygous or heterozygous deletion or monosomy. As expected, homozygous deletion was always associated with loss of p16 expression but otherwise we found little correlation between copy number and p16 immunoreactivity. Notably, in our study 27% of chordomas with loss of p16 protein expression exhibited a normal diploid *CDKN2A* copy number status, a finding also reported by Le *et al* 6 in two of 18 cases. Furthermore, 30 of 147 (20%) cases with complete loss of p16 protein expression showed heterozygous loss and 18 of 147 (12%) showed monosomy. This raises the question of the mechanism by which the loss of protein occurs.

The frequent loss of p16 protein expression not associated with homozygous loss of *CDKN2A* could not be explained by a variety of mechanisms that we have investigated. Specifically, promoter DNA hypermethylation, one of the most common mechanisms implicated in the silencing of *CDKN2A* in tumours, was not found to account for this, confirming the work of others who reported that only one of 15 chordoma cases tested had definitive evidence of *CDKN2A* promoter methylation 6. Only one case, a clival chordoma, showed higher levels of DNA methylation for one of the probes at the promoter region. An interesting finding is that this case showed negativity for INI‐1, a feature that is found in poorly differentiated chordomas, which are known to exhibit a methylation profile different from conventional chordomas 34. Furthermore, with only one in 35 cases revealing copy number neutral LOH, a common copy number alteration caused by uniparental disomy and usually associated with homozygous mutations, homozygous deletions or alterations in cancer‐promoting imprinted genes 35, 36, this mechanism is unlikely to explain the loss of p16 protein in the absence of homozygous deletion. Indeed, the copy number neutral LOH identified in one chordoma was associated with p16 positivity in the absence of SNVs or indels, a finding rarely reported in other cancers 37.

We pursued two other major lines of enquiry in an attempt to explain the mechanism by which p16 protein could be lost. First, we analysed whether the SNP rs11515 was associated with *CDKN2A* expression because of the known association in melanoma, sporadic colorectal, skin, bladder, cervical, breast cancer and glioblastoma 28; however, we failed to detect any association. Second, we tested the expression of miRNAs which were previously shown to control p16 protein expression. MiR‐24‐2, a negative regulator of p16, blocks p16 translation in keratinocytes and chondrocytes 30, 31. Increased expression of miR‐10b‐5p correlates with reduced expression of its target genes including *CDKN2A* in renal papillary carcinoma and glioma 32. miR‐125b is a known tumour suppressor gene that blocks translation of a number of transcripts involved in the control of cell proliferation in various cancers 33, but a convincing association of miR‐125 and p16 expression has not been reported, with the exception of a study showing that hsa‐miR‐125b exhibited significant negative correlations with *CDKN2A* expression in glioblastoma multiforme 38. In this study we excluded an association between p16 and the expression of these miRNAs.

In view of the difficulty in explaining the loss of p16 protein by promoter hypermethylation and other selected epigenetic events, even though this has only been undertaken using a candidate approach, a number of reasons argue for a post transcriptional regulatory mechanism to explain the loss of protein in the presence of a retained allele as determined by FISH. It is otherwise difficult to explain how comparable levels of *CDKN2A* could be detected in chordomas with and without protein expression. Although *CDKN2A* mRNA would be expressed by non‐neoplastic cells in chordomas sample, this cannot be the case in the three chordoma cell lines in which mRNA was detected despite the loss of protein expression in the presence of monosomy for chromosome 9. Only one cell line (JHC7) was immunoreactive for p16 in the presence of monosomy, whereas the other three showed *CDKN2A* homozygous deletion and absence of protein expression.

The median life expectancy of patients with chordoma is 7 years 1. However, for individuals, it can range from months to more than 25 years (case 143), hence having a prognostic biomarker such as p16 IHC, which could be undertaken easily, would be valuable for patients and clinicians. However, retention of p16 expression cannot be reliably employed to predict less aggressive behaviour in chordoma, because it may be heterogeneously expressed, and detection is dependent on tumour sampling. Furthermore, as immunoreactivity is lost in 53% of our samples and others have reported an even greater percentage, we do not consider that it is useful to employ p16 IHC as a prognostic marker. However, p16 protein expression could be valuable for stratification of patients for the purposes of a clinical trial using a CDK4/6 inhibitor, in which it would be advisable to assess multiple areas 15. Finally, as much of the expression of p16 is controlled at a post transcriptional level, using FISH as a biomarker to determine protein expression provides limited information and is not recommended in a clinical setting.

## Author contributions statement

AMF and LC conceived the study. AMF supervised the study. LC, NE, IU, LL and HY carried out the investigation. PL carried out bioinformatic analysis. RT, AMF and DL provided clinical data. AMF, SB, JP, NP, RT and FA curated samples. LC, AMF, IU and NP wrote the paper with input from other co‐authors.

## Supporting information


**Supplementary materials and methods**

**Figure S1.** Patterns of *CDKN2A* copy number aberrations using whole genome sequencing data
**Figure S2.** Lack of *CDKN2A* promoter DNA methylation in chordoma samples and expression of p16, p14 and ANRIL in chordoma cases dependent on rs11515 SNP genotype
**Figure S3.** Heterogeneous expression of p16 in chordoma samples

**Table S1.** p16/*CDKN2A* assessed by IHC, genomic alterations and DNA methylation in 10 chordoma cases also analysed by whole genome sequencing
**Table S2.** p16 heterogeneity in chordoma

## Data Availability

Infinium Methylation array data of chordoma and U2OS cell lines have been deposited in the National Center for Biotechnology Information GEO database under GEO accession number (GSE139410).
