# Supplementary material for: Frequent alterations in p16/CDKN2A identified by immunohistochemistry and FISH in chordoma
Source: J Pathol Clin Res. 2020 Jan 8;6(2):113–23. doi: 10.1002/cjp2.156 (PMC7164370; doi:10.1002/cjp2.156)
Supplement: Supplementary file 1 — Supplementary materials and methods [file CJP2-6-113-s002.docx]

**Frequent alterations in p16/CDKN2A identified by immunohistochemistry and FISH in chordoma**

Cottone L *et al*. *J Pathol Clin Res* DOI: 10.1002/cjp2.156

**Supplementary materials and methods**

The reference number refers to the main paper

***Quantitative real-time PCR (qPCR*)**

RNA was extracted from frozen cell line pellets or tissue curls using the miRNeasy Mini Kit (Qiagen, Manchester, Lancashire, UK) and transcribed into cDNA using the High Capacity cDNA Reverse Transcription Kit (Applied Biosystems, Thermo Fisher Scientific, Loughborough, Leicestershire, UK) according to the manufacturers’ instructions.

Quantitative real-time PCR (qPCR) was performed as previously reported using Fast SYBR® Green MasterMix (Applied Biosystems) according to the manufacturer’s instructions [16].

The primers used were as follows: *p16/CDKN2A* forward: 5’- gcaccagaggcagtaaccat -3’, reverse: 5’- ttctttcaatcggggatgtc-3’; *GAPDH* forward: 5’- ACACCCACTCCTCCACCTTTG -3’, reverse: 5’- CATACCAGGAAATGAGCTTGACAA -3’.

For analysis, the means of cycle thresholds (Ct) (n=3) were calculated. Based on the deltaCt between *CDKN2A* and *GAPDH*, the fold increase of *CDKN2A* over *GAPDH* was determined [=POWER(2,-deltaCt)].

***miRNA expression***

Total RNA was extracted from frozen chordoma curls using the miRNeasy Mini Kit (Qiagen, Hilden, Germany). Equal amounts of RNA were reverse transcribed into cDNA in a single reaction step using the miRCURY LNA™ Universal RT according to the manufacturer's instructions. The cDNA synthesis control (UniSp6) was added in the reverse transcription reaction giving the opportunity to evaluate the RT reaction. Quantitative PCR (qPCR) was performed using the Exilent SYBR Green mastermix according to the manufacturer's instructions. LNA PCR primer set for spike control (UniSp6 v2), one candidate endogenous miRNA (miR-103a-3p), miR-10, miR-24-3p, and miR-125b-5p were used. Results were analysed using the 2-ΔCT method normalised to mir-103a expression.

***DNA methylation***

DNA was extracted from chordoma (UCH1, UM-Chor, UCH7, MUG-Chor) and U2OS cell lines using a DNA Mini Kit (Qiagen) according to the manufacturer’s instructions. Between 0.5μg and 1μg of DNA was run on the Infinium Methylation EPIC array (Illumina) or 450K Infinium Human Methylation array (Illumina). Data were assembled using BeadStudio (Illumina) and processed using Minfi^1^ using the functional normalisation protocol. Beta values were downloaded from the Cancer Genome Atlas for cancers other than chordoma.
