# Supplementary material for: Frequent alterations in p16/CDKN2A identified by immunohistochemistry and FISH in chordoma
Source: J Pathol Clin Res. 2020 Jan 8;6(2):113–23. doi: 10.1002/cjp2.156 (PMC7164370; doi:10.1002/cjp2.156)
Supplement: Supplementary file 2 — Figure S1. Patterns of CDKN2A copy number aberrations using whole genome sequencing data Figure S2. Lack of CDKN2A promoter DNA methylation in chordoma samples and expression of p16, p14 and ANRIL in chordoma cases dependent on rs11515 SNP genotype Figure S3. Heterogeneous expression of p16 in chordoma samples [file CJP2-6-113-s001.docx]

**Frequent alterations in p16/CDKN2A identified by immunohistochemistry and FISH in chordoma**

Cottone L *et al*. *J Pathol Clin Res* DOI: 10.1002/cjp2.156

**Supplementary Figures**

The reference number refers to the main paper.

**
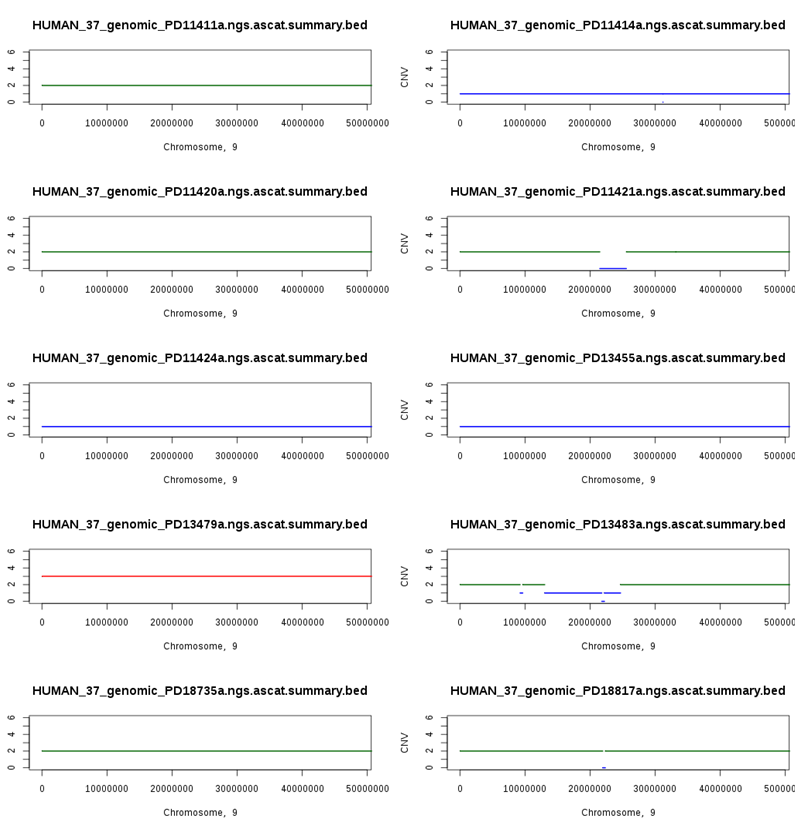
Figure S1. Patterns of *CDKN2A* copy number aberrations using whole genome sequencing data.**

*CDKN2A* copy number showed a broad spectrum of alterations in ten chordoma cases: three samples showed entire chromosome 9p losses (blue), three cases showed heterozygous or homozygous deletions (Green/blue), one case showed chromosome 9p polysomy (red) and three cases had a normal copy number status (green).

**Figure S2. Lack of *CDKN2A* promoter DNA methylation in chordoma samples and expression of p16, p14 and ANRIL in chordoma cases dependent on rs11515 SNP genotype.** (A) Heatmap of the β-value for promoter-associated Infinium probes. The promoter region of *CDKN2A* is unmethylated in all primary human chordomas (n=35) and in the sacral chordoma cell lines (UCH1, UCH7) but it is highly methylated in the clival chordoma cell line UM-Chor. The osteosarcoma cell line U2OS, which showed high promoter methylation, consistent with results previously reported in the literature [16], was used as control. (B-D) Gene expression was analysed in ten chordomas by RNA-sequencing. No differential expression was observed on p16, p14 or ANRIL dependent on the rs11515 genotype.

**Figure S3. Heterogeneous expression of p16 in chordoma samples.**

Images of two chordoma cases (case 1: A-D and case 2: E-H) showing heterogeneous expression of p16: H&E (A and E, 4x objective magnification), focal p16 immunoreactivity at lower (B and F, 4x objective magnification) and higher magnification of the dashed area (C and G, 10x objective magnification). p16 in a negative area showing internal positive control (D and H, 4x objective magnification).
