# Supplementary material for: Frequent alterations in p16/CDKN2A identified by immunohistochemistry and FISH in chordoma
Source: J Pathol Clin Res. 2020 Jan 8;6(2):113–23. doi: 10.1002/cjp2.156 (PMC7164370; doi:10.1002/cjp2.156)
Supplement: Supplementary file 3 — Table S1. p16/CDKN2A assessed by IHC, genomic alterations and DNA methylation in 10 chordoma cases also analysed by whole genome sequencing Table S2. p16 heterogeneity in chordoma [file CJP2-6-113-s003.docx]

**Frequent alterations in p16/CDKN2A identified by immunohistochemistry and FISH in chordoma**

Cottone L *et al*. *J Pathol Clin Res* DOI: 10.1002/cjp2.156

**Supplementary Tables**

**Table S1.** p16/CDKN2A assessed by IHC, genomic alterations and DNA methylation in 10 chordoma cases also analysed by whole genome sequencing.

| Study ID | Ethnicity* | *CDKN2A* FISH | p16 IHC | SNVs/indels | *CDKN2A* (p16^INK^) promoter methylation | G500 SNP genotype | *TBXT* FISH |
| --- | --- | --- | --- | --- | --- | --- | --- |
| 17 | White british | Disomy | p16 positive | No | Hypomethylated | CC |  |
| 26 | White british | Disomy | p16 negative | No | Hypomethylated | CC |  |
| 52 | White british | Monosomy | p16 negative | No | Hypomethylated | CC | Amplified |
| 133 | White (others) | Homo deletion | p16 negative | No | Hypomethylated | CC | Amplified |
| 60 | White british | Disomy | p16 positive | No | Hypomethylated | CG | Amplified |
| 62 | White british | Disomy | p16 positive | No | Hypomethylated | CG |  |
| 21 | White british | Homo deletion | p16 negative | No | Hypomethylated | CG |  |
| 132 | Asian indian | Hetero deletion | p16 negative | No | Hypomethylated | CG |  |
| 89 | White (others) | Polysomy | p16 positive | No | Hypomethylated | CG |  |
| 53 | Chinese | Monosomy | p16 negative | No | Hypomethylated | CG |  |

The whole genome data analysis supported the FISH results. FISH and IHC results generated from full tissue sections. *, ethnicity self-assigned.

**Table S2*.*** p16 heterogeneity in chordoma

| Study unique ID | Stage | Number of sections analysed | p16 immunoreactivity |
| --- | --- | --- | --- |
| 39 | Primary | 2 | 1 with focal immunoreactivity |
|  | LR1 | 2 | 1 with focal immunoreactivity |
| 95 | Primary | 10 | 9 negative, 1 with focal immunoreactivity |
| 61 | Primary | 7 | 6 negative, 1 with focal immunoreactivity |
|  | LR1 | 1 | All negative |
|  | LR2 | 2 | All negative |
| 90 | Primary | 8 | All negative |
| 56 | Primary | 6 | All negative |
| 22 | Primary | 6 | All negative |

LR1-LR2, first to second local recurrence
